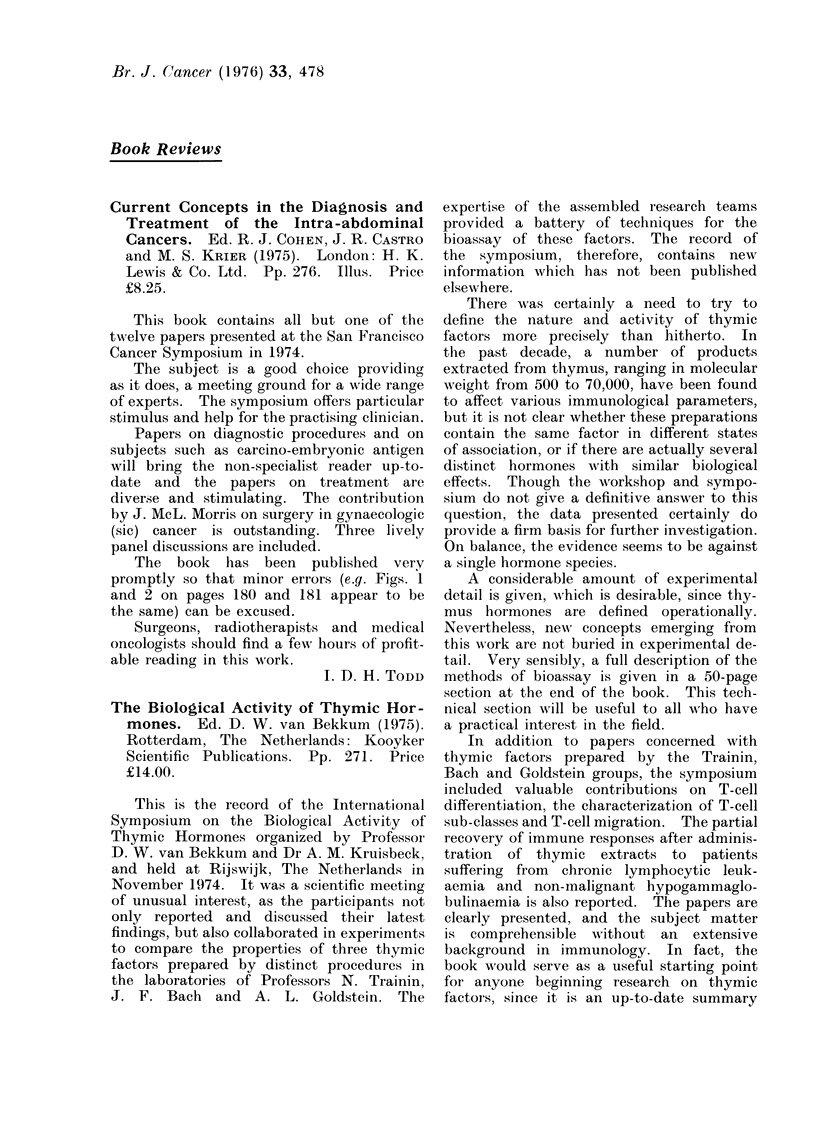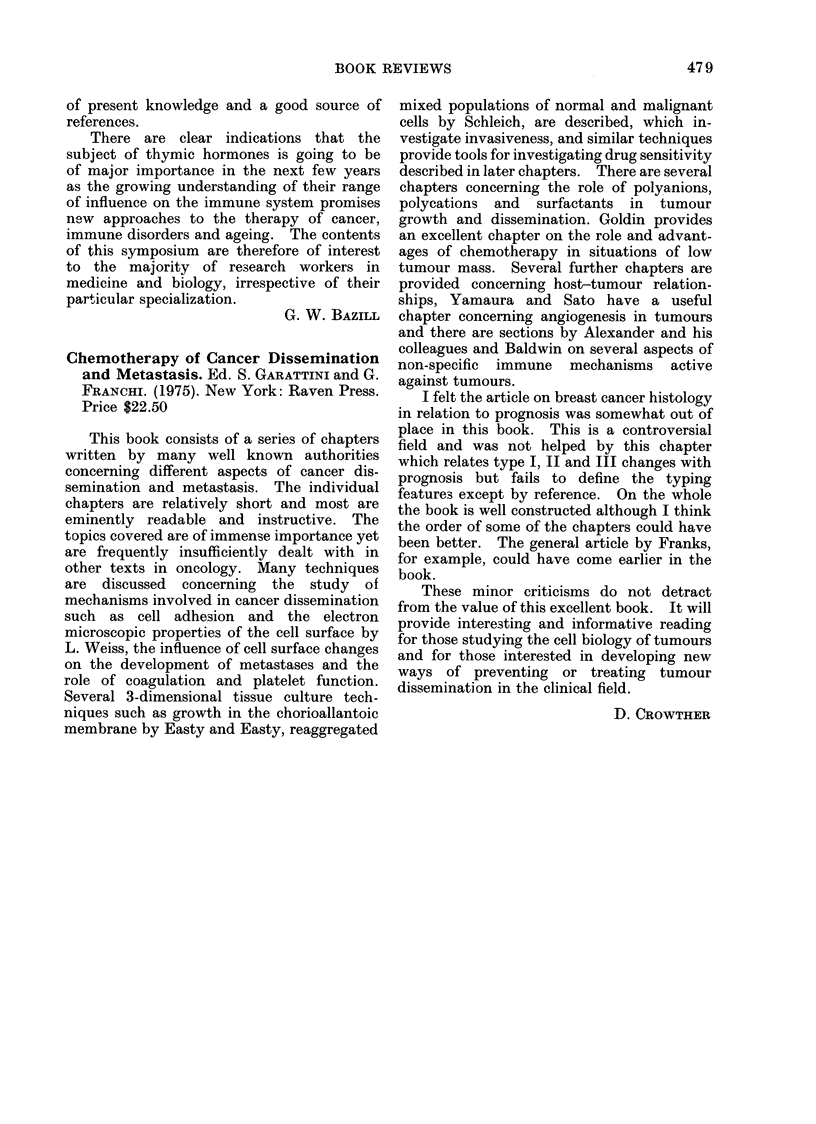# The Biological Activity of Thymic Hormones

**Published:** 1976-04

**Authors:** G. W. Bazill


**The Biological Activity of Thymic Hormones.** Ed. D. W. van Bekkum (1975). Rotterdam, The Netherlands: Kooyker Scientific Publications. Pp. 271. Price £14.00.

This is the record of the International Symposium on the Biological Activity of Thymic Hormones organized by Professor D. W. van Bekkum and Dr A. M. Kruisbeck, and held at Rijswijk, The Netherlands in November 1974. It was a scientific meeting of unusual interest, as the participants not only reported and discussed their latest findings, but also collaborated in experiments to compare the properties of three thymic factors prepared by distinct procedures in the laboratories of Professors N. Trainin, J. F. Bach and A. L. Goldstein. The expertise of the assembled research teams provided a battery of techniques for the bioassay of these factors. The record of the symposium, therefore, contains new information which has not been published elsewhere.

There was certainly a need to try to define the nature and activity of thymic factors more precisely than hitherto. In the past decade, a number of products extracted from thymus, ranging in molecular weight from 500 to 70,000, have been found to affect various immunological parameters, but it is not clear whether these preparations contain the same factor in different states of association, or if there are actually several distinct hormones with similar biological effects. Though the workshop and symposium do not give a definitive answer to this question, the data presented certainly do provide a firm basis for further investigation. On balance, the evidence seems to be against a single hormone species.

A considerable amount of experimental detail is given, which is desirable, since thymus hormones are defined operationally. Nevertheless, new concepts emerging from this work are not buried in experimental detail. Very sensibly, a full description of the methods of bioassay is given in a 50-page section at the end of the book. This technical section will be useful to all who have a practical interest in the field.

In addition to papers concerned with thymic factors prepared by the Trainin, Bach and Goldstein groups, the symposium included valuable contributions on T-cell differentiation, the characterization of T-cell sub-classes and T-cell migration. The partial recovery of immune responses after administration of thymic extracts to patients suffering from chronic lymphocytic leukaemia and non-malignant hypogammaglobulinaemia is also reported. The papers are clearly presented, and the subject matter is comprehensible without an extensive background in immunology. In fact, the book would serve as a useful starting point for anyone beginning research on thymic factors, since it is an up-to-date summary of present knowledge and a good source of references.

There are clear indications that the subject of thymic hormones is going to be of major importance in the next few years as the growing understanding of their range of influence on the immune system promises new approaches to the therapy of cancer, immune disorders and ageing. The contents of this symposium are therefore of interest to the majority of research workers in medicine and biology, irrespective of their particular specialization.

G. W. BAZILL